# Nephrogenic Adenoma of the Prostatic Urethra Mimicking Prostatic and Bladder Carcinomas

**DOI:** 10.7759/cureus.35998

**Published:** 2023-03-10

**Authors:** Maria C Beeter, Tajammul Fazili, Yunshin A Yeh

**Affiliations:** 1 Pathology and Translational Pathobiology, Louisiana State University Health Sciences Center, Shreveport, USA; 2 Urology, Overton Brooks Veterans Health Administration (VA) Medical Center, Shreveport, USA; 3 Pathology and Laboratory Medicine, Overton Brooks Veterans Health Administration (VA) Medical Center, Shreveport, USA; 4 Urology, Louisiana State University Health Sciences Center, Shreveport, USA

**Keywords:** prostatic urethra, clear cell adenocarcinoma, urothelial cell carcinoma, prostatic adenocarcinoma, nephrogenic adenoma

## Abstract

A nephrogenic adenoma is a benign lesion consisting of the proliferation of tubules and glands in the urinary tract. The lesion, thought to be originated from renal tubules, is commonly seen in the urinary bladder. Microscopically, nephrogenic adenoma is composed of a proliferation of small tubules and microcysts encircled by a narrow rim of basement membrane-like hyaline material. There are tubules and microcysts lined by atrophic to undulating hobnail-appearing epithelial cells with bland nuclei and pale eosinophilic to clear cytoplasm. Focal cellular atypia characterized by somewhat coarse chromatin and prominent nucleoli may be present. The stroma is edematous and reveals a granulation tissue-like appearance. By immunohistochemical staining, nephrogenic adenoma is positive for PAX-2, PAX-8, P504S (α-methylacyl-CoA racemase), pan cytokeratin AE1/AE3, CK7, CAM5.2, epithelial membrane antigen (EMA), CD10, and napsin A. Occasionally the lesions are incidentally encountered in the prostatic urethra, which may lead to a misdiagnosis as prostatic adenocarcinoma, clear cell adenocarcinoma or urothelial carcinoma of the urinary bladder. Herein we present a case of nephrogenic adenoma which has been incidentally found in a transurethral resection of a prostate specimen for the management of benign prostatic hypertrophy. The evaluation of morphology, immunohistochemistry, and differential diagnoses have also been discussed.

## Introduction

A nephrogenic adenoma is a benign entity consisting of the proliferation of tubular and glandular-like structures in the urinary tract and occurs most frequently in the urinary bladder. The incidence is unknown. Sometimes, the lesion can be found in the urethra and the ureter [1]. Patients' ages range from 15 to 94 (average 52) years [1]. Males are more commonly affected than females, with a ratio of two to one [1]. Most cases are seen in adults, but children can also be affected [2]. Nephrogenic adenomas are usually small, but some lesions have measured up to 7cm. The lesion is more commonly found in the urinary bladder (55%). Other sites of occurrence, such as the urethra (41%) and ureter (4%), have also been reported [1]. Nephrogenic adenoma mimics prostatic adenocarcinoma if it occurs in the prostatic urethra [3,4]. The lesion can also mimic clear cell adenocarcinoma of the urinary bladder [5,6]. Many affected patients present with a clinical history of urinary tract injury, including trauma and surgery [5]. Clinical presentations often include gross hematuria, microhematuria, urinary incontinence, and dysuria [7]. Urethrocystoscopic examination reveals solitary or multiple small nodules with various gross appearances of gray-tan, polypoid, and granular lesions [2]. The clinical manifestations, gross appearance, and microscopic features mimicking prostatic and bladder cancers can pose diagnostic challenges for urologists and pathologists [8,9]. Herein we present a case of nephrogenic adenoma of the prostatic urethra. Histopathological features, immunohistochemical staining patterns, differential diagnoses, and potential diagnostic pitfalls are also discussed.

## Case presentation

A 72-year-old African-American male presented with urinary frequency, urgency, and urinary incontinence. A cystoscopic examination performed in 2019 revealed trilobar prostatic enlargement with a prominent median lobe. The patient was lost to follow-up for two years and was seen in the Urology Clinic, where a transurethral resection of the prostate (TURP) was performed in June 2021. Pathological examination of the transurethral resection specimen revealed benign prostatic tissue with predominantly stromal hyperplasia and focal chronic inflammation. However, the patient had progressive symptoms of dysuria, frequency, incomplete bladder emptying, and intermittent gross hematuria. One year later, abdominal and pelvic CT scans were performed and revealed moderate hydroureteronephrosis in bilateral kidneys, diffusely abnormal distension of both ureters, an indented urinary bladder by an enlarged prostate, and a 6 cm lobulated mass suggestive of bladder cancer. Cystoscopic examination showed small vessels with bleeding in the prostatic urethra, particularly proximal to the verumontanum. There was a very large prostate with contact bleeding. It was difficult to locate the left ureteral orifice, and the right ureteral orifice was identified. The bladder mucosal surface appeared trabeculated and revealed no significant abnormalities. Furthermore, the bladder washing cytology showed no high-grade urothelial carcinoma cells. Subsequently, a TURP was performed in September 2022.

**Figure 1:**
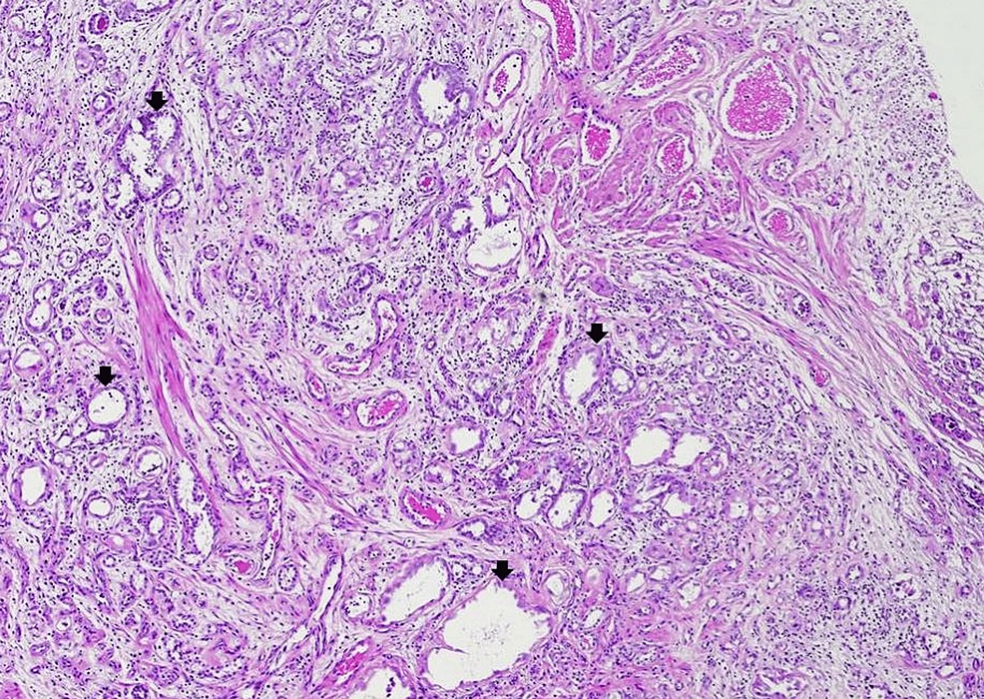
Nephrogenic adenoma The transurethral resection of the prostate specimen shows a proliferation of small tubules/glands and microcyts in a pseudoinfiltrative pattern. The lamina propria has a granulation tissue-like edematous appearance (hematoxylin-eosin, original magnification x40).

**Figure 2:**
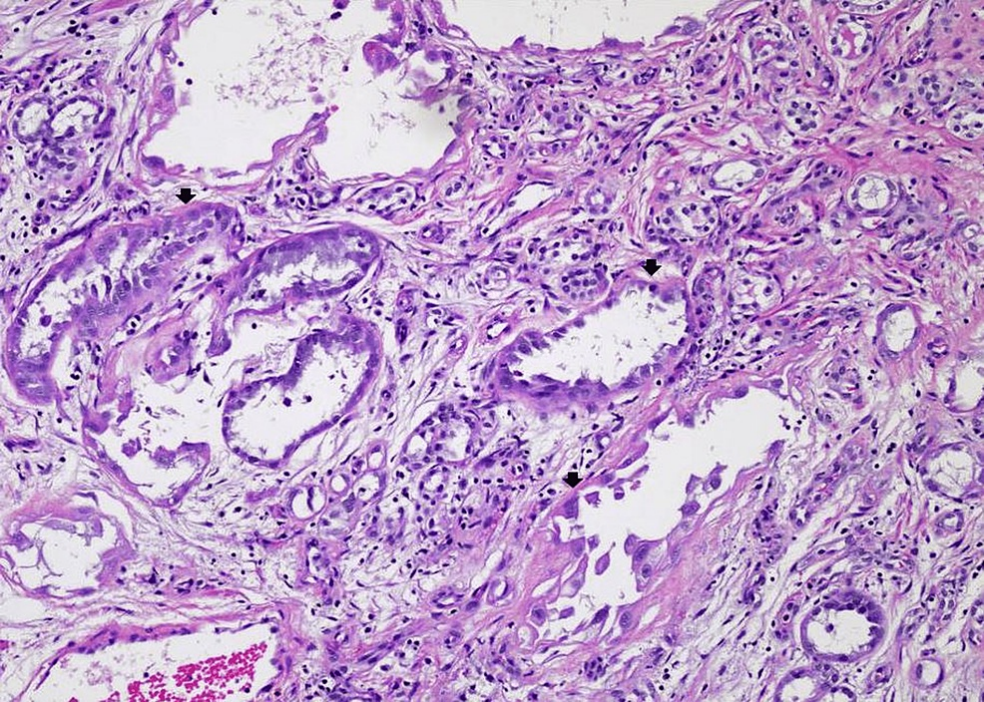
Nephrogenic adenoma Many tubulocystic structures are encircled by a narrow rim of basement membrane-like hyalinized material. Microcystic glands are lined by undulating flattened to low cuboidal cells with a "hobnail" appearance. Focal atypical cells characterized by somewhat coarse chromatin, prominent nucleoli, and vacuolated cytoplasm are demonstrated; however, no mitotic figures are present (hematoxylin-eosin, original magnification x100).

**Figure 3:**
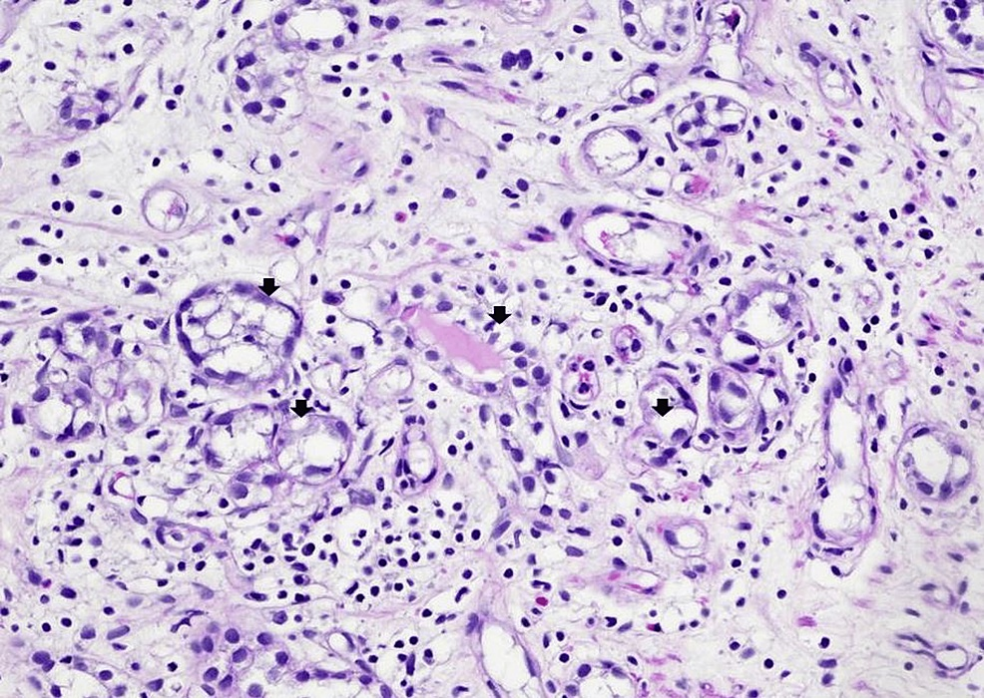
Nephrogenic adenoma The tubules and microcysts are lined by atrophic to low cuboidal epithelial cells with bland nuclei and pale eosinophilic to clear cytoplasm. The stroma is markedly edematous and consists of chronic inflammatory infiltrates with rare eosinophils (hematoxylin-eosin, original magnification x200).

**Figure 4:**
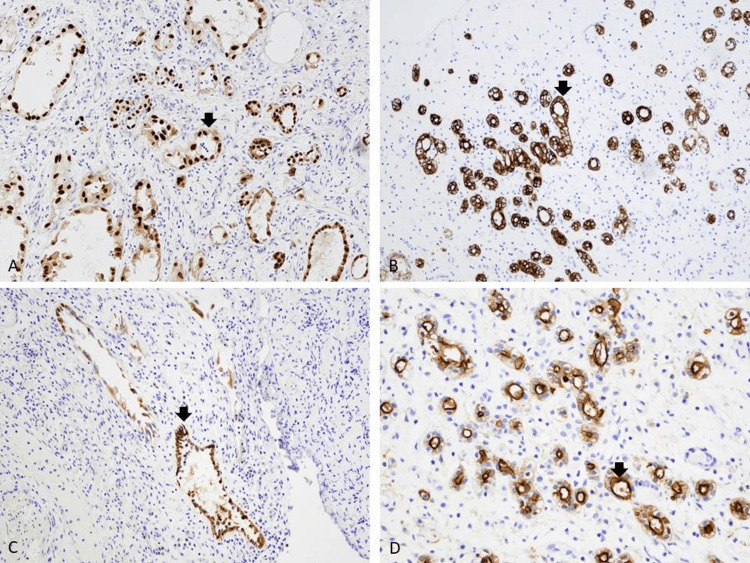
Immunohistochemical stains (A) The tubules/glands and microcysts lining epithelial cells show diffusely positive nuclear staining with PAX-8 immunomarker. (B) CK7 immunohistochemical stain is diffusely positive in the epithelial cells. (C) Focal microcystic epithelial cells express P504S (α-methylacyl-CoA-racemase, AMACR) protein. (D) The tubular cells show a diffuse reactivity to epithelial membrane antigen (EMA) immunomarker (immunoperoxidase stains; A-C, original magnification x100; D, original magnification x200).

Histopathological examination of the transurethral resection of the prostate specimen revealed a proliferation of small tubules and microcysts (Figure 1). Many tubulocystic structures are encircled by a narrow rim of basement membrane-like hyaline material (Figure 2). Other areas of the lesion displayed tubules and microcysts lined by atrophic to cuboidal epithelial cells with bland nuclei and pale eosinophilic to clear cytoplasm (Figure 3). There are microcystic glands lined by undulating flattened lining cells and hobnail-appearing cells (Figure 2). Focal cellular atypia characterized by somewhat coarse chromatin and prominent nucleoli is noted (Figure 2). Mitotic activities are absent. These proliferating tubules and microcystic structures are embedded in a background of granulation tissue-like edematous stroma with mixed inflammatory infiltrate (Figures 2, 3). Immunohistochemical stains showed that the tubular epithelial cells are positively stained with PAX-8, CK7, P504S (α-methylacyl-CoA-racemase, AMACR focally), and epithelial membrane antigen (EMA) (Figures 4A-4D). Other immunomarkers, including NKx3.1, prostate-specific antigen (PSA), prostate specific alkaline phosphatase (PSAP), GATA3, and high molecular weight cytokeratin, are stained negative in this lesion (not shown). The uninvolved area of the prostatic chips showed predominantly benign fibromuscular and stromal hyperplasia, ureteritis cystica of the prostatic urethra, and focal acute and chronic inflammation. Based on the results of morphology and immunohistochemical evaluation, a diagnosis of nephrogenic adenoma is rendered.

## Discussion

Macroscopically, nephrogenic adenoma appears as a papillary, polypoid, exophytic, sessile, or flat lesion [8,9]. Sessile and flat lesions may resemble urothelial carcinoma-in-situ on urethrocystoscopy [9]. Microscopic examination of nephrogenic adenoma shows a broad spectrum of histologic features, of which the tubular and glandular configuration represents the most frequent pattern [1]. Other patterns of nephrogenic adenoma include papillary proliferation of fibrous cores covered by a single layer of benign flattened to low cuboidal cells with a "hobnail" appearance. Cystic structures lined by atrophic epithelium and containing intraluminal mucinous materials are also commonly seen [1,3]. The cystic glands and tubules are encircled by a rim of basement membrane-like hyalinized sheath and are lined by undulating epithelial cells with degenerative/reactive atypical nuclei, prominent nucleoli, and eosinophilic to clear cytoplasm [1,8]. Nonetheless, many cases reveal a mixed pattern with exophytic, tubular/glandular, and microcystic features. Occasionally, signet ring-like cells with intraluminal mucin are present, and a thyroid-like pattern may be noted [3,8]. A mitotic figure is extremely rare. The small tubules and cystically dilated glands are embedded in an edematous granulation tissue-like stroma with acute and chronic inflammation [8]. Desmoplastic changes are unequivocally absent. Rarely, small foci of solid growth with clear cell change may be present in nephrogenic adenoma; however, solid sheets of proliferation, pronounced cellular atypia, and brisk mitotic activities should not be observed [1]. By immunohistochemical staining, nephrogenic adenoma is positive for PAX-2, PAX-8, P504S (α-methylacyl-CoA-racemase, AMACR), pan cytokeratin AE1/AE3, CK7, CAM5.2, EMA, CD10, napsin A, and high-molecular-weight cytokeratin 34bE12 (patchy staining) [9]. In some occasions, PSA and PSAP immunomarkers may be focally and weakly positive [9]. Immunostains NKx3.1, GATA-3, p63, and high molecular weight cytokeratin are negative [9]. A low proliferative index with Ki-67 ranging from 0% to 5% of positively stained nuclei is noted.

For many years, nephrogenic adenoma has been proposed to represent the proliferation of mesonephric remnants or metaplastic growth of the bladder urothelium [3,4]. This assumption is based on the association of nephrogenic adenoma with bladder irritation, previous surgery, and unusual healing response to an injurious process [3,4]. Another study has shown that nephrogenic adenoma is associated with previous intravesical treatment with bacillus Calmette-Guérin for urothelial carcinoma of the bladder [7]. Nephrogenic adenoma has also been suggested to be originated from the renal tubular cells through a shedding and seeding process [8]. As a consequence, the shed renal tubular cells implant and grow into a site of the injured urothelium. This hypothesis is supported by the findings that nephrogenic adenomas in renal-transplant recipients' bladder are derived from the renal tubular cells of the donor instead of the metaplastic proliferation of the recipient's urothelium [8]. This pathogenic shedding and seeding process may contribute to the random distribution and an infiltrative-like growth pattern of the lesion. Other studies suggest that nephrogenic adenoma underwent metaplastic transformation proliferates as a response to local mucosal stimuli [1,2,10]. Regardless of its etiology, the haphazard proliferation of small round and hollow or solid tubules seen in nephrogenic adenoma raises the differential diagnoses of prostatic adenocarcinoma, clear cell adenocarcinoma of the urinary bladder, and invasive urothelial carcinoma including microcystic variant and villoglandular differentiation [3,5,6].

Nephrogenic adenoma has the potential morphologic overlap with invasive prostatic adenocarcinoma with a higher Gleason grade [3]. The crowded tubular and glandular growth composed of a monolayer of monotonous nuclei and without basal cell layer of nephrogenic adenoma can closely mimic prostatic adenocarcinoma [3]. In addition, the signet ring-like cells with intraluminal mucin present a diagnostic challenge in the interpretation of bladder and urethral biopsies and transurethral resection specimens [3,9]. However, the "hobnail" and undulating appearance of epithelial lining cells in nephrogenic adenoma is extremely rare or absent in prostatic adenocarcinoma. In addition, pronounced cellular atypia, including nucleomegaly, hyperchromasia, and prominent nucleoli commonly seen in prostatic adenocarcinoma glands, are not present in nephrogenic adenoma. Furthermore, nephrogenic adenoma usually has a hypervascular, inflammatory, and edematous stroma that is rarely observed in prostatic adenocarcinoma [3,9]. In difficult cases, differential immunomarkers may be applied to distinguish these entities. PAX-2 and PAX-8 immunomarkers are commonly positive in nephrogenic adenoma [11]. While PSA and PSAP may be focally or weakly positive, NKx3.1 immunomarker, which consistently stained positive in prostatic adenocarcinoma, is negative in nephrogenic adenoma [12,13]. Therefore, the positivity of PAX-8 and negativity of NKx3.1, PSA, and PSAP in our case refute a diagnosis of prostatic adenocarcinoma. 

Nephrogenic adenoma, with its common papillary and tubulocystic patterns, is a close mimicker of clear cell adenocarcinoma of the urinary bladder [5,6]. The exophytic growth of nephrogenic adenoma is covered by a single layer of monotonous hobnail-appearing cells, whereas clear cell adenocarcinoma is composed of proliferating and anastomosing papillae covered by a monolayer of cuboidal cells with eosinophilic to clear cytoplasm and marked nuclear pleomorphism [5,6]. In addition, there are many areas of epithelial stratification and cellular tufting in clear cell adenocarcinoma. Nonetheless, the eosinophilic to clear cytoplasm of the adenocarcinoma cells may not be a significant differential feature since diffuse and solid growth pattern with clear cell change has also been described in nephrogenic adenoma [1]. Immunohistochemically, clear cell adenocarcinoma expresses PAX-2, PAX-8, p53, CA125, carcinoembryonic antigen (CEA), CK7, and P504S (α-methylacyl-CoA-racemase, AMACR). NKx3.1, PSA, PSAP, CK20, estrogen receptor (ER), and progesterone receptor (PR) immunomarkers are negative [5,6,10]. Brisk mitotic activity with a proliferating index Ki-67 of 10% to 80% compared to a low proliferation rate of 0% to 5% in nephrogenic adenoma is noted (6). In our case, there is no complex branching papillary growth. Additionally, the glandular lining epithelial cells have bland nuclei with focal mild reactive nuclear atypia. The epithelial cells are negatively stained by immunomarkers p53 and CEA. Moreover, the epithelial cells are mitotically inactive with a low proliferative index Ki-67 of approximately 2%. Taken together, these features exclude a differential diagnosis of clear cell adenocarcinoma. It has been suggested that nephrogenic adenoma may progress to clear cell adenocarcinoma. Comparative genomic hybridization analysis has shown that relative loss of 4q33pter and 9q, relative gain of 8q and 1q41qter, and chromosome 17 alterations occur in both nephrogenic adenoma and clear cell adenocarcinoma [14]. However, the missense mutation p53 gene that occurred in clear cell adenocarcinoma is not present in nephrogenic adenoma [14]. Despite its clonal alteration similar to that of clear cell adenocarcinoma, nephrogenic adenoma has not been considered as a premalignant lesion.

Nephrogenic adenoma with papillary and tubulocystic architectures can also mimic an invasive urothelial carcinoma with glandular differentiation [9]. Generally, the invasive high-grade urothelial carcinoma has irregular and haphazard infiltrating borders and various degrees of complex anastomosis [9,15]. The tumor cells show marked atypia characterized by nuclear enlargement, hyperchromasia, or vesicular nuclei with coarse chromatin, irregular nuclear membranes, and prominent nucleoli. Increased mitotic figures are present. These features of malignant transformation, however, are not present in nephrogenic adenoma. On other occasions, the nested or microcystic variant of urothelial carcinoma with deceptively bland nuclei may resemble the monotonous benign cells in nephrogenic adenoma, which may lead to a misdiagnosis of invasive urothelial carcinoma [15]. However, increasing cellular atypia may be appreciated in deeper portions of invasive tumor cells in a nested and tubular variant of urothelial carcinoma [15]. Moreover, the presence of muscularis propria invasion can be considered a definitive diagnostic feature of invasive urothelial carcinoma [15]. Sometimes it seems impossible to render a definitive diagnosis in small superficial bladder biopsies. In these occasions, an immunohistochemical study may be helpful and provide some diagnostic clues. The urothelial carcinoma cells express GATA-3, p63, and uroplakin proteins and are negative for PAX-2 and PAX-8 immunomarkers [16]. In our case, the tubular and glandular cells are positively stained with PAX-8 and are negative for GATA-3 and p63 immunomarkers. As a result, these findings are consistent with nephrogenic adenoma.

The vast majority of nephrogenic adenomas are treated with transurethral resection. The management not only allows an accurate histopathologic diagnosis but also provides symptomatic relief. Despite its benign and reactive nature, nephrogenic adenomas have a high risk of recurrences [17]. A recurrence rate of up to 63% has been reported [17,18]. Postoperative long-term follow-up with frequent cystoscopy is essential due to its high recurrence rate. Most of the recurrent lesions can also be treated endoscopically to provide relief of symptoms [17,18].

## Conclusions

A nephrogenic adenoma is a benign lesion of the bladder and is sometimes found in the prostatic urethra. Clinical manifestations include hematuria, dysuria, and urinary frequency. In regard to the pathogenesis, theories including growth of the mesonephric remnants, metaplastic proliferation of renal tubular cells, and shedding and seeding of the proliferating renal tubular cells have been proposed. Although there is no clear consensus on the pathogenesis of nephrogenic adenoma, the proposal of renal tubular metaplasia may better explain those lesions that occur in distal sites, in particular the prostatic urethra presented in our case. The clinical significance of nephrogenic adenoma is the possibility of misdiagnosis as a high-grade prostatic adenocarcinoma or carcinoma of the urinary bladder. Attention to the differential morphologic features and the applications of specific immunohistochemical stains could be useful in the distinction of these entities. A nephrogenic adenoma is not considered or proved to be a premalignant lesion despite several case studies proposing its progression to adenocarcinoma. However, the benign lesion has a high rate of recurrence, and the recurrent lesion can also be treated endoscopically. 
